# Supplementary material for: Genomic release-recapture experiment in the wild reveals within-generation polygenic selection in stickleback fish
Source: Nat Commun. 2020 Apr 21;11:1928. doi: 10.1038/s41467-020-15657-3 (PMC7174299; doi:10.1038/s41467-020-15657-3)
Supplement: Supplementary file 1 — Supplementary Information [file 41467_2020_15657_MOESM1_ESM.pdf]

Supplementary Figures

Genomic release-recapture experiment in the wild reveals within-generation polygenic selection in stickleback fish

Laurentino et al.

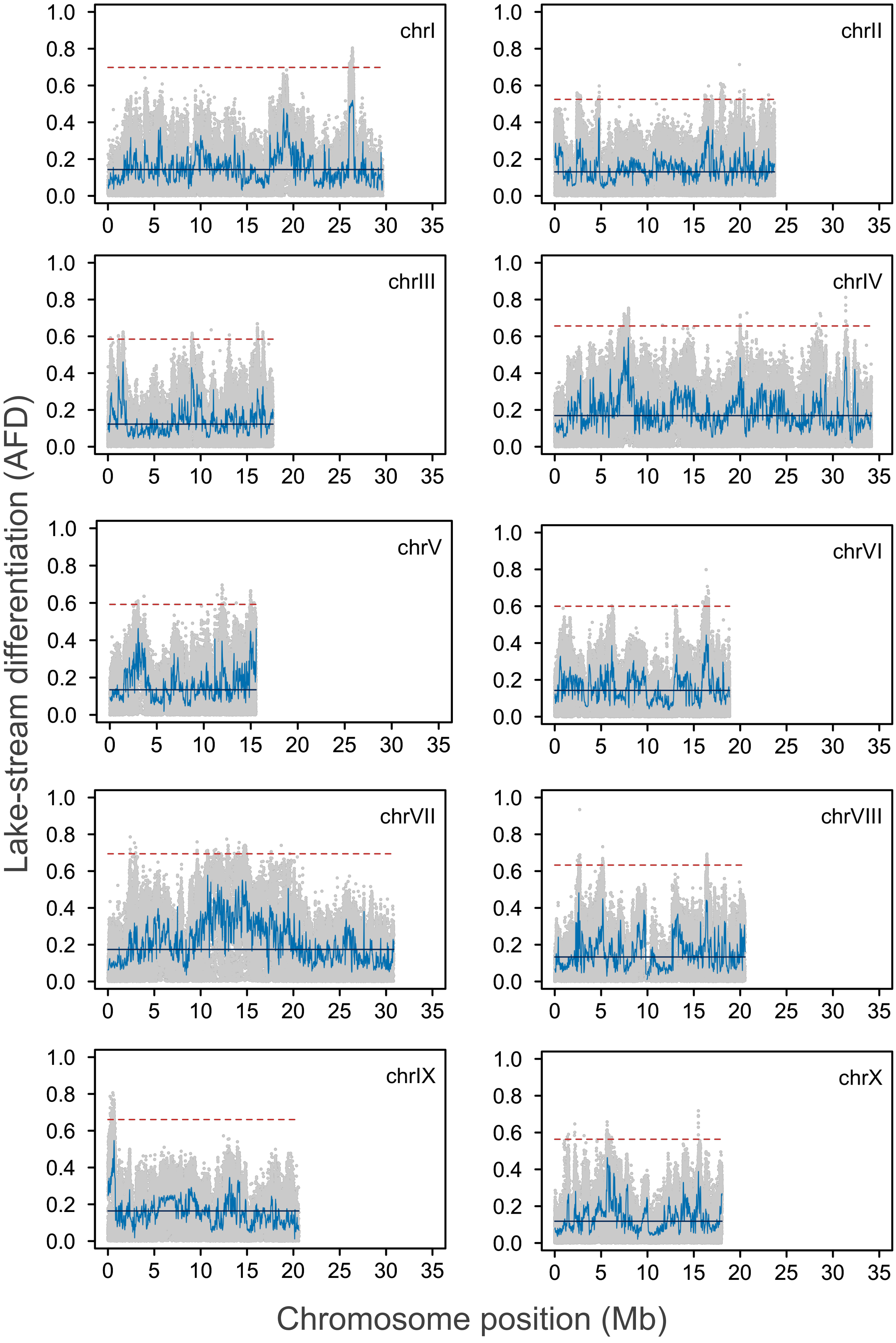
chrI
chrII
chrIII
chrIV
chrV
chrVI
chrVII
chrVIII
chrIX
chrX
1.0
0.8
0.6
0.4
0.2
0.0
0
5
10
15
20
25
30
35
Lake-stream differentiation (AFD)
Chromosome position (Mb)

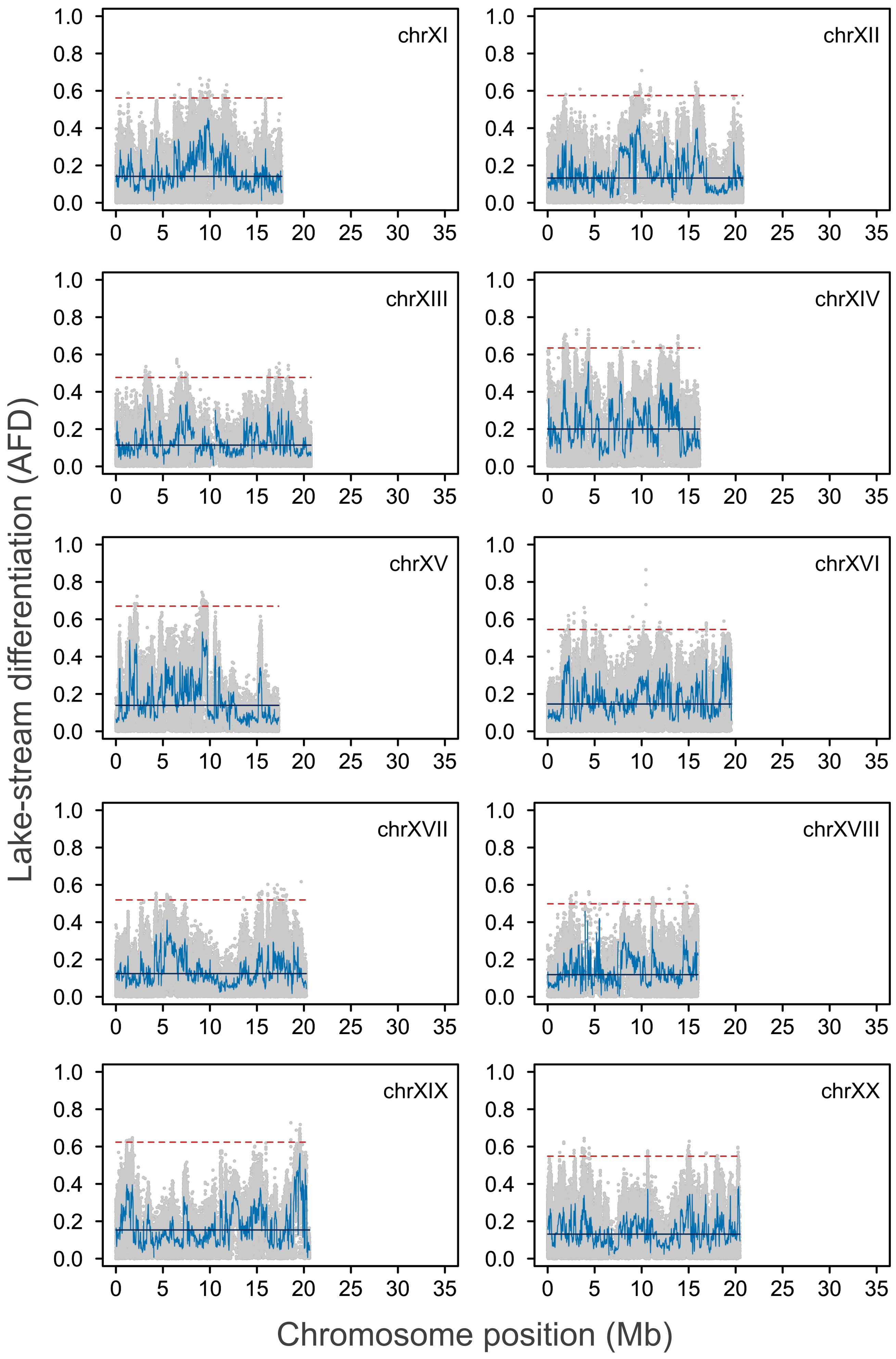
chrXI
chrXII
chrXIII
chrXIV
chrXV
chrXVI
chrXVII
chrXVIII
chrXIX
chrXX
1.0
0.8
0.6
0.4
0.2
0.0
0
5
10
15
20
25
30
35
Lake-stream differentiation (AFD)
Chromosome position (Mb)

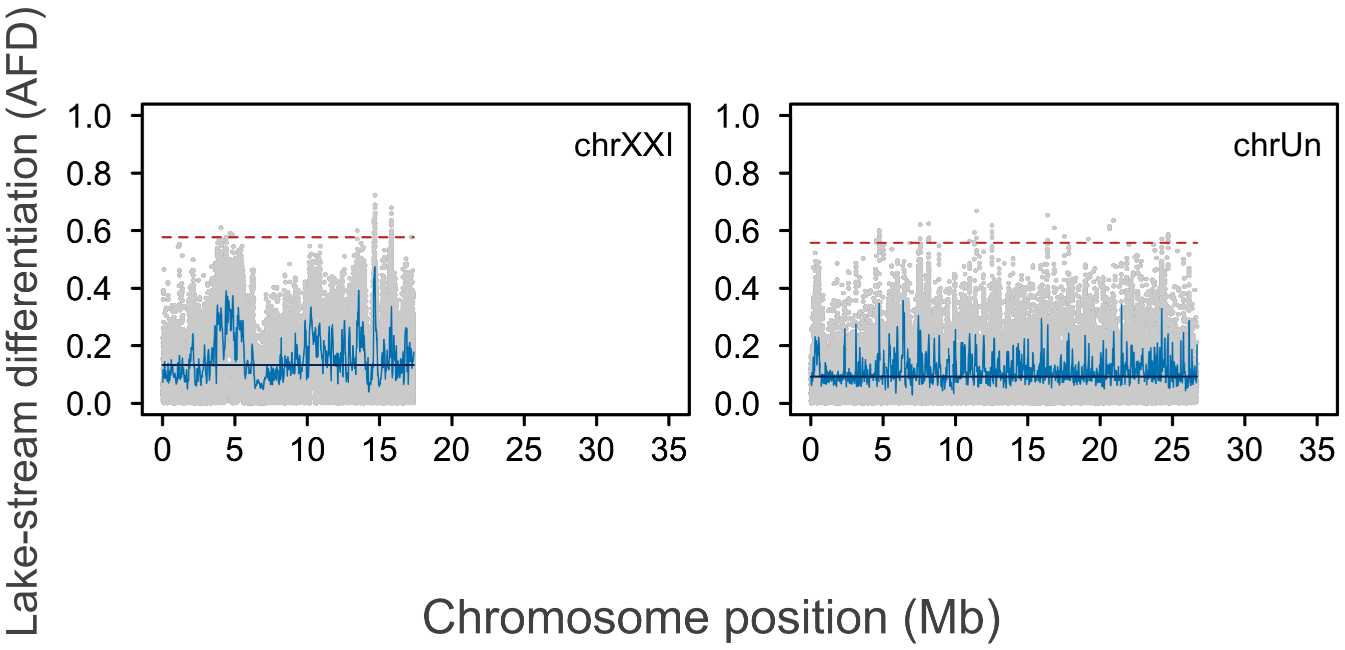

Supplementary Figure 1. **Genomic differentiation between the natural lake and stream populations**. Profiles of the absolute allele frequency difference (AFD) are shown for all chromosomes, with the gray dots representing individual single-nucleotide polymorphisms (SNPs) and the black lines indicating chromosome-specific median values. The blue curves show differentiation smoothed by averaging AFD across SNPs for 40 kb sliding windows with 20 kb overlap. The dashed red lines give the upper 0.1 percentile of the chromosome-specific AFD distribution used as a threshold to delimit candidate genome regions under divergent lake-stream selection.

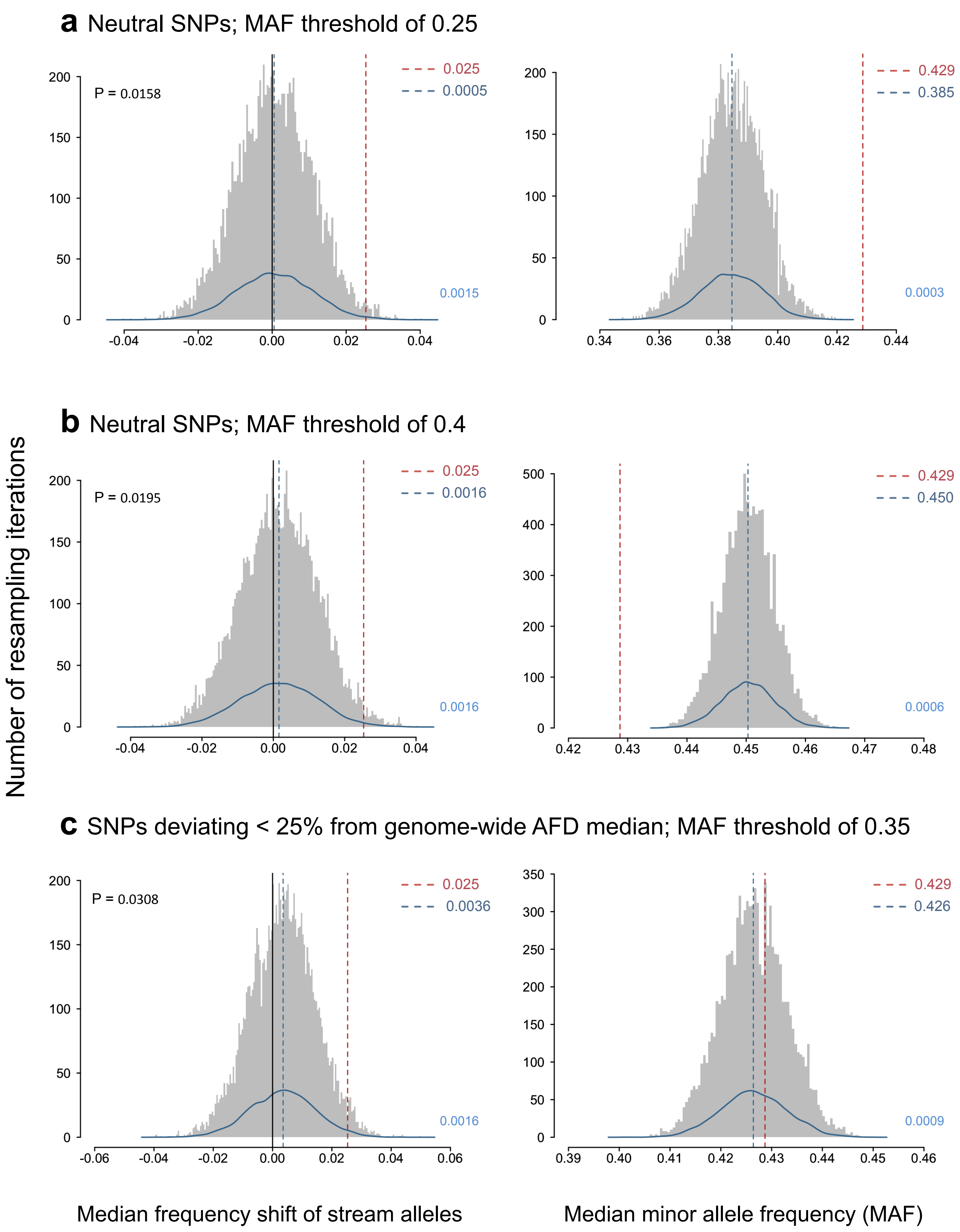
a Neutral SNPs; MAF threshold of 0.25
P = 0.0158
0.025
0.0005
0.0015
0.429
0.385
0.0003
b Neutral SNPs; MAF threshold of 0.4
P = 0.0195
0.025
0.0016
0.0016
0.429
0.450
0.0006
c SNPs deviating < 25% from genome-wide AFD median; MAF threshold of 0.35
P = 0.0308
0.025
0.0036
0.0016
0.429
0.426
0.0009
Number of resampling iterations
Median frequency shift of stream alleles
Median minor allele frequency (MAF)

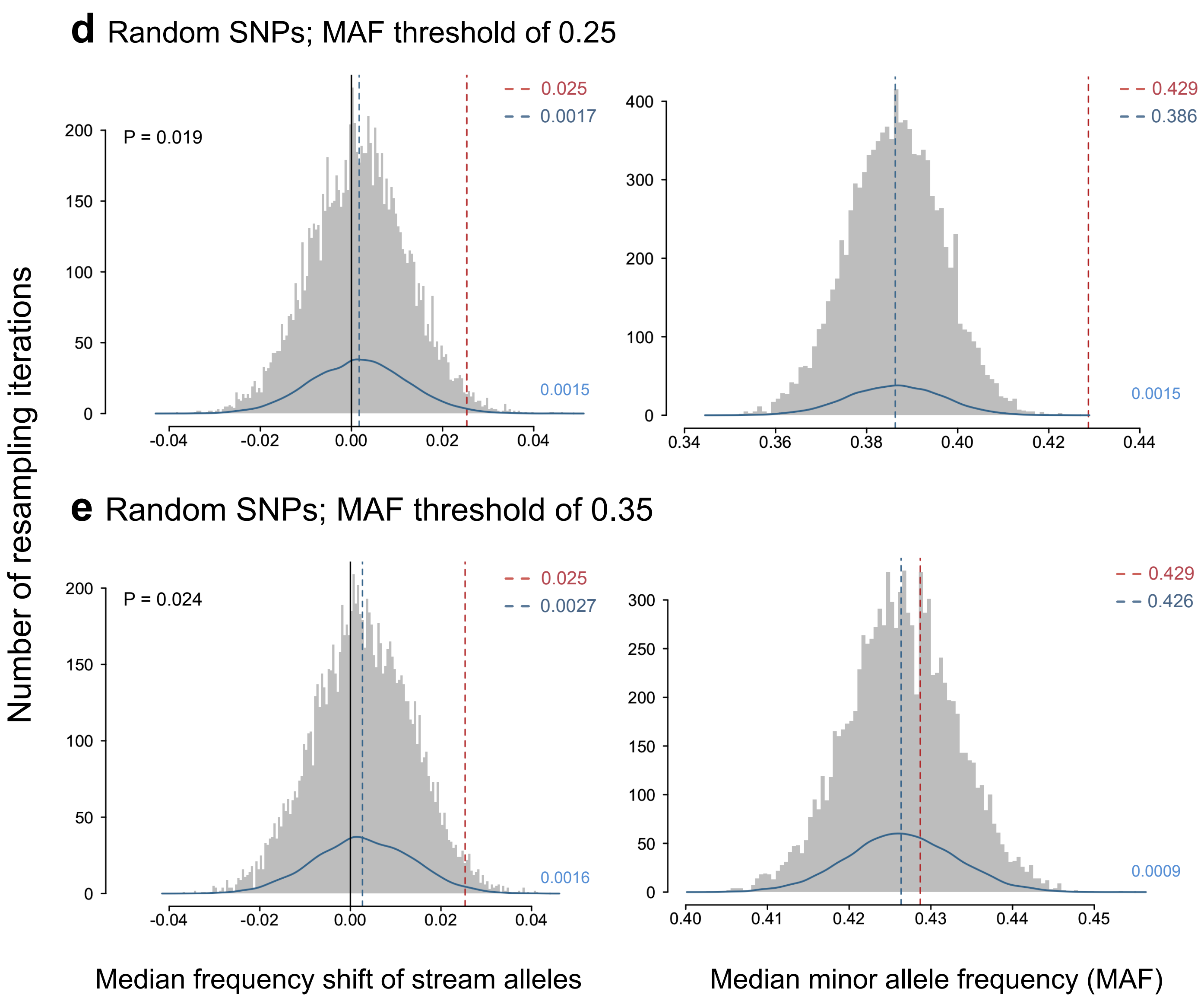

Supplementary Figure 2. **Robustness checks for the resampling approach used to evaluate the experimental allele frequency shifts observed at the target SNPs against a genome-wide baseline.** Different resampling protocols were implemented (always based on 9999 iterations), varying in the SNPs considered for resampling, and in their minimum minor allele frequency (MAF) exhibited in the reference sample. For each resampling data set, we present in the left panel the distribution of the median allele frequency shift across the 126 resampled SNPs, adopting the graphing conventions of Figure 4 (in addition, the two-tailed P value for the median shift across the target SNPs given the focal resampling distribution is indicated and band width values are presented in blue at the top-right of desity lines). The right panels characterize the distribution of the median MAF across the resampled SNPs, following the graphing conventions of Supplementary Fig. 6. **a** and **b** are based on the neutral SNPs also used for the main analysis presented in the paper (0 - 0.1 AFD window in the natural lake-stream population comparison), but with a less stringent (0.25) and a more stringent (0.4) MAF threshold applied. In **c**, the standard MAF threshold of 0.35 was used, but the SNP panel for resampling contained only those SNPs deviating no more than 25% in both directions from the genome-wide median AFD in the natural population comparison (corresponding to an AFD window of 0.1 – 0.17). In **d** and **e**, the SNPs for resampling were drawn completely at random with respect to the magnitude of differentiation in the natural population comparison, applying two different MAF thresholds. Note that whatever the details underlying the resamling protocols, the shifts observed across the target SNPs during the field experiment remain extreme as compared to the resamling distributions, thus highlighting the robustness of our inference of experimental genomic evolution driven by natural selection. We further note that under most resampling conditions, the grand median allele frequency shifts are slightly greater than zero. We hypothesize that this pattern reflects genomically widespread hitchhiking along with the alleles under selection.

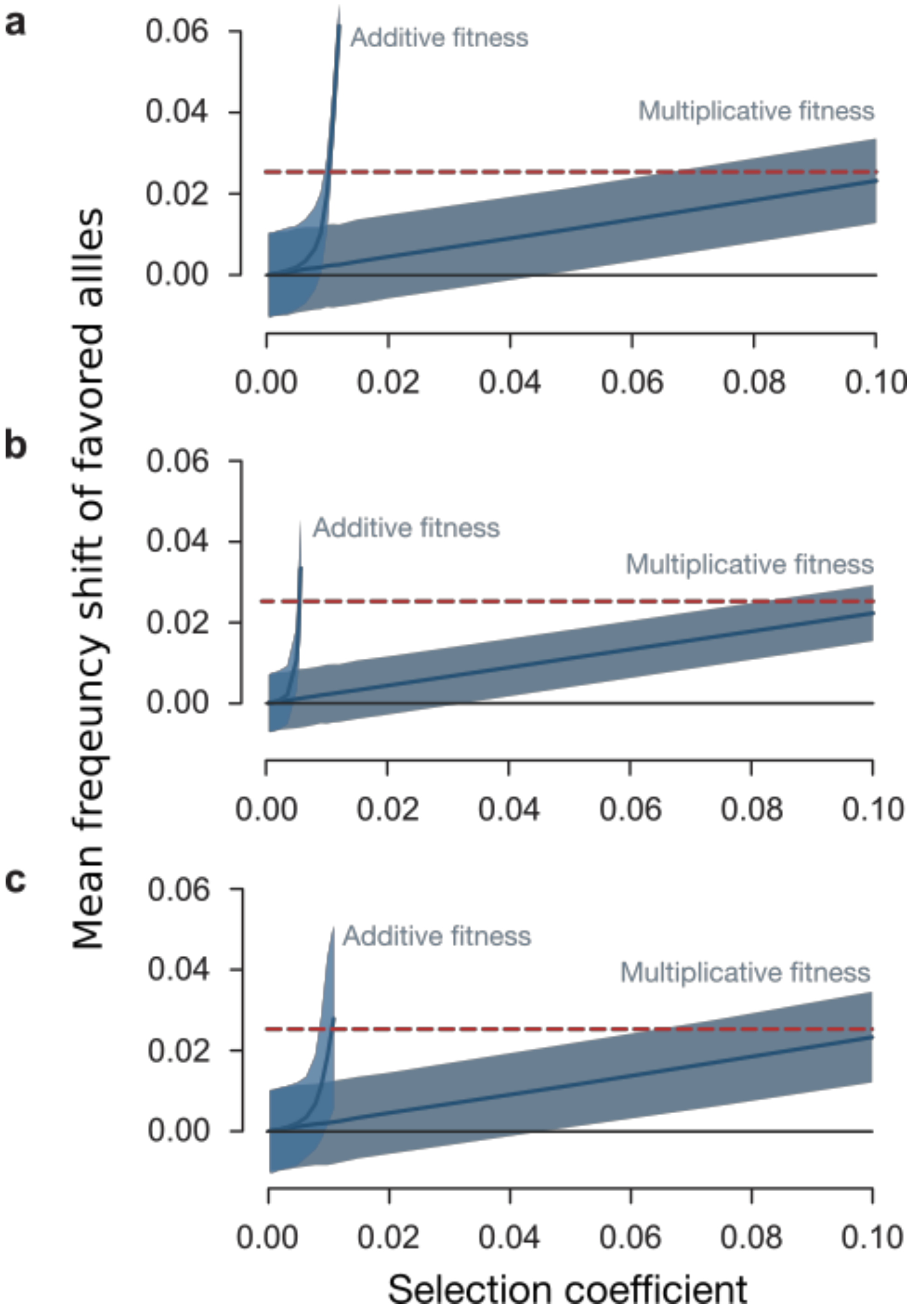

Supplementary Figure 3. Allele frequency shifts in relation to selection coefficients during simulated directional polygenic selection over a single generation, for three supplementary modeling conditions. The simulation and graphing conventions are like those underlying Figure 5a, but the data were generated by assuming **a** a larger initial population size (3000), **b** a greater number of loci under selection (200), and **c** selection coefficients not being identical among loci but drawn from an exponential distribution.

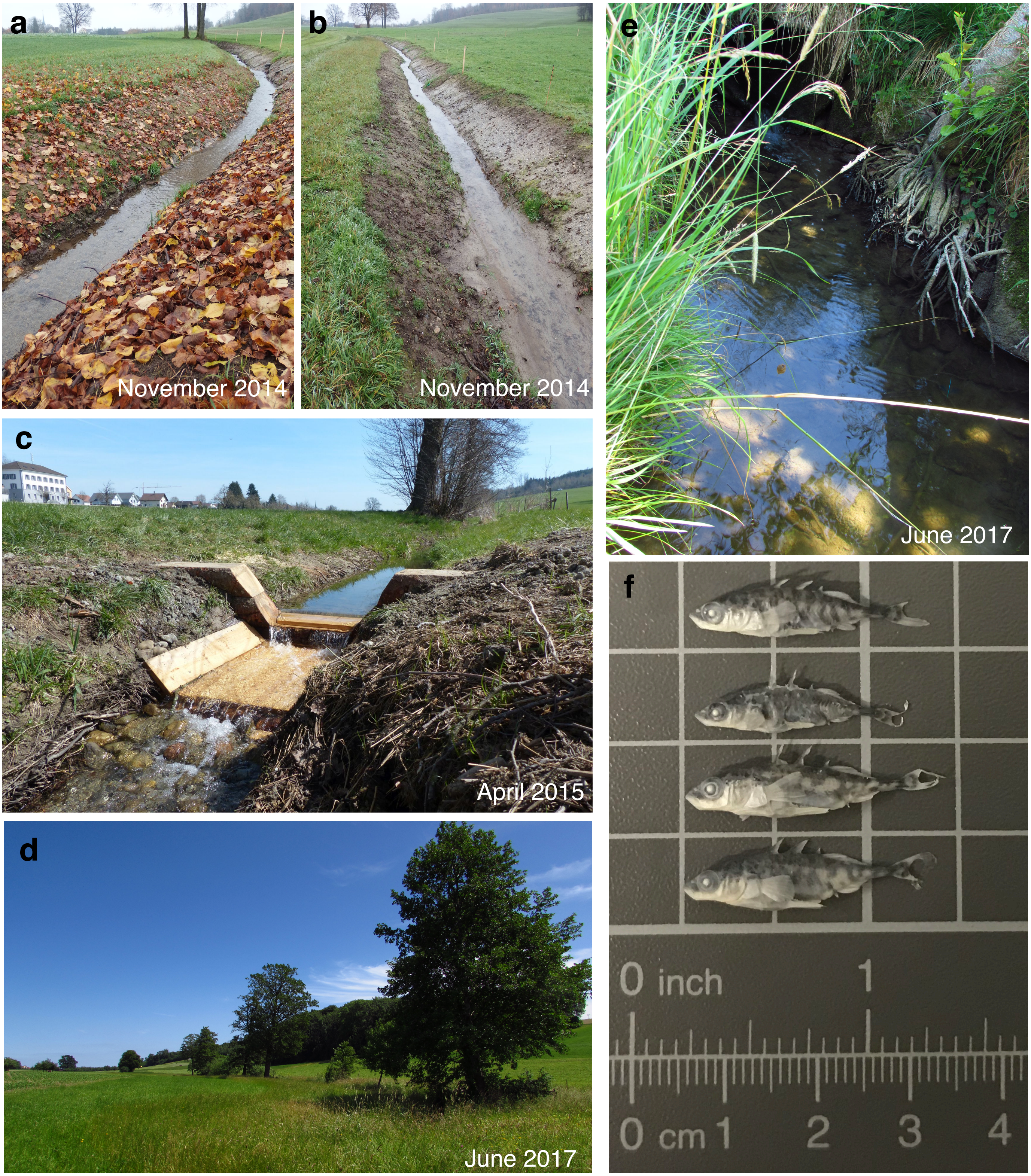

Supplementary Figure 4 - Release-recapture experiment stream and released F2 hybrids. Photos of the stream (47°32'44.156" N, 9°13'23.631" E) one year prior to the experiment onset (November 2014). The stream initially had limited water level (**a** and **b**), to increase water volume and carrying capacity dams were built in April 2015 (**c**). This guaranteed a stream section of c. 50 m of suitable stickleback habitat (**d**, **e**). The F2 hybrids (**f**, four randomly picked individuals from the reference sample; tail tissue was used for DNA extraction) were released on September 2015. Photo credit: TGL and DB.

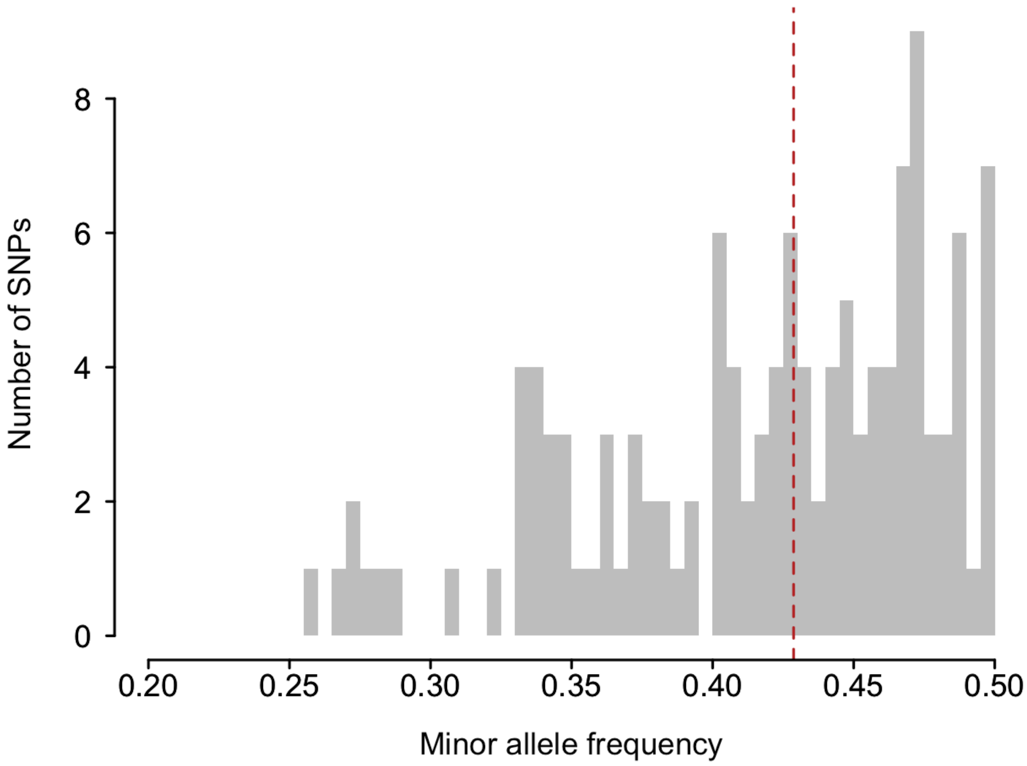

Supplementary Figure 5. Minor allele frequency (MAF) distribution of the target SNPs in the reference sample. The target SNP were required to satisfy a MAF threshold of 0.25 in the reference sample. The observed median MAF, indicated by the dashed red line, was 0.429.

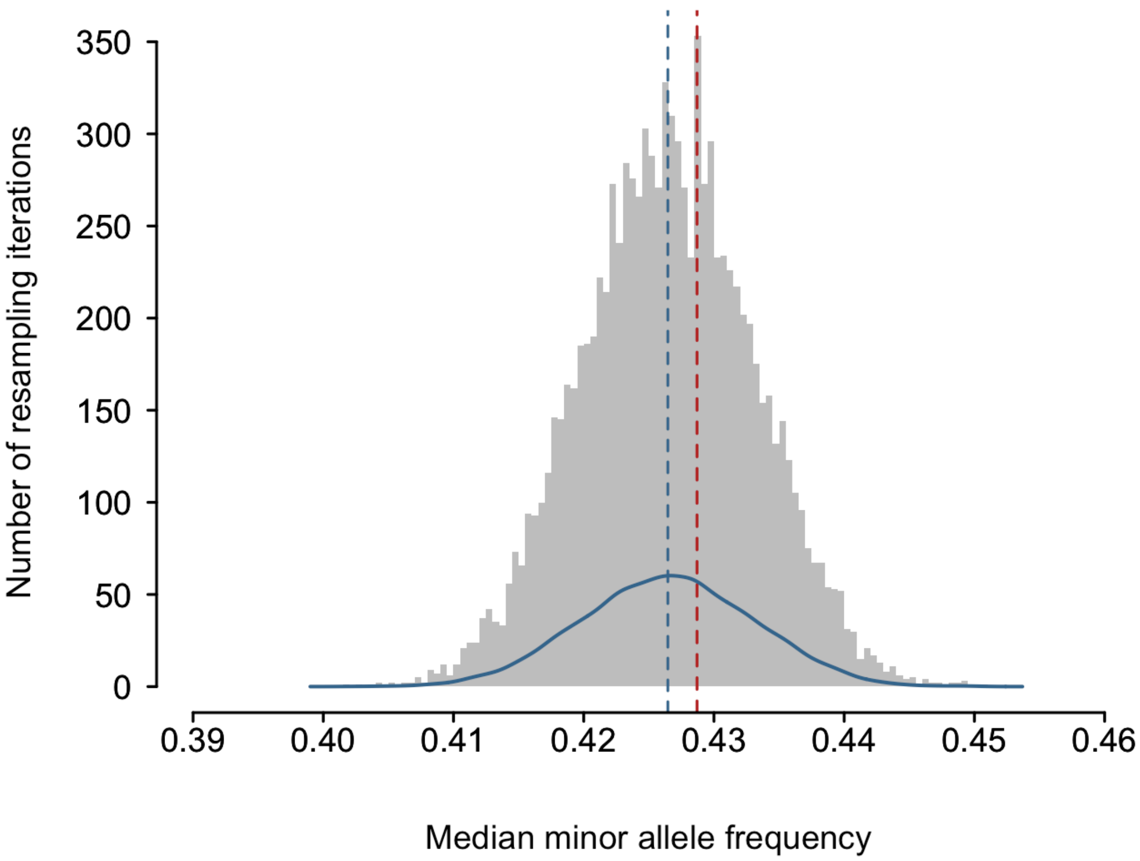

Supplementary Figure 6. Minor allele frequency (MAF) distribution among the resampled sets of neutral SNPs used to evaluate the allele frequency shifts observed at the target SNPs. Shown is the distribution across the 9999 resampling iterations of the median MAF across the 126 neutral SNPs drawn in each iteration. The neutral SNPs were here MAF-filtered using a threshold of 0.35 in the reference sample, and hence coincide with the SNPs underlying the neutral allele frequency shift distribution presented in Figure 4, The blue curve visualizes the MAF distribution kernel density-smoothed (bandwidth: 1.4), and the grand median (0.426) is indicated by the dashed blue line. The dashed red line indicates the median MAF observed across the 126 target SNPs (0.429; Figure S3).
