# Supplementary material for: Genomic release-recapture experiment in the wild reveals within-generation polygenic selection in stickleback fish
Source: Nat Commun. 2020 Apr 21;11:1928. doi: 10.1038/s41467-020-15657-3 (PMC7174299; doi:10.1038/s41467-020-15657-3)
Supplement: Supplementary file 3 — Reporting Summary [file 41467_2020_15657_MOESM3_ESM.pdf]

nature research

Corresponding author(s): telma.laurentino@unibas.ch
daniel.berner@unibas.ch

Last updated by author(s): Mar 7, 2020

Reporting Summary

Nature Research wishes to improve the reproducibility of the work that we publish. This form provides structure for consistency and transparency in reporting. For further information on Nature Research policies, see Authors & Referees and the Editorial Policy Checklist .

Statistics

For all statistical analyses, confirm that the following items are present in the figure legend, table legend, main text, or Methods section.

n/a	Confirmed	
☐	☒	The exact sample size (*n*) for each experimental group/condition, given as a discrete number and unit of measurement
☐	☒	A statement on whether measurements were taken from distinct samples or whether the same sample was measured repeatedly
☐	☒	The statistical test(s) used AND whether they are one- or two-sided<br>*Only common tests should be described solely by name; describe more complex techniques in the Methods section.*
☒	☐	A description of all covariates tested
☐	☒	A description of any assumptions or corrections, such as tests of normality and adjustment for multiple comparisons
☐	☒	A full description of the statistical parameters including central tendency (e.g. means) or other basic estimates (e.g. regression coefficient) AND variation (e.g. standard deviation) or associated estimates of uncertainty (e.g. confidence intervals)
☐	☒	For null hypothesis testing, the test statistic (e.g. *F*, *t*, *r*) with confidence intervals, effect sizes, degrees of freedom and *P* value noted<br>*Give P values as exact values whenever suitable.*
☒	☐	For Bayesian analysis, information on the choice of priors and Markov chain Monte Carlo settings
☒	☐	For hierarchical and complex designs, identification of the appropriate level for tests and full reporting of outcomes
☐	☒	Estimates of effect sizes (e.g. Cohen's *d*, Pearson's *r*), indicating how they were calculated

Our web collection on statistics for biologists contains articles on many of the points above.

Software and code

Policy information about availability of computer code

Data collection	For almost all genomic analyses and simulations, we used custom R codes, all of which are provided in the Supplementary Software file.
Data analysis	The only ready-made software used is the aligner (Novoalign; version 3.03.00 run on Linux 2.6). The full alignment settings are specified in the Supplementary Software file. All analytical code was scripted by ourselves and is available, with details on packages and respective versions, in the Supplementary Software file.

For manuscripts utilizing custom algorithms or software that are central to the research but not yet described in published literature, software must be made available to editors/reviewers. We strongly encourage code deposition in a community repository (e.g. GitHub). See the Nature Research guidelines for submitting code & software for further information.

Data

Policy information about availability of data

All manuscripts must include a data availability statement. This statement should provide the following information, where applicable:
- Accession codes, unique identifiers, or web links for publicly available datasets
- A list of figures that have associated raw data
- A description of any restrictions on data availability

All raw whole-genome sequencing data are available from the NCBI sequence read archive (SRA) under the study number SRP222265 and the accession numbers listed by sample in the Supplementary Data 1. All input files needed for full replication of the present study are provided as Supplementary Data 2-6.

Field-specific reporting

Please select the one below that is the best fit for your research. If you are not sure, read the appropriate sections before making your selection.

Ecological, evolutionary & environmental sciences study design

All studies must disclose on these points even when the disclosure is negative.

Study description	Study of natural selection in action in lake and stream stickleback fish. Uses a genome scan based on poolSeq whole-genome sequence data from two natural populations (lake and stream, N = 240 and 229 individuals) to identify candidate targets of selection. Next, F2 hybrid individuals derived from the natural populations are generated (196 crosses), and 3000 individuals released into natural stream habitat. Finally, the whole-genome sequence data from N = 37 survivors are compared against reference individuals (N = 510) from the initial F2 hybrid population to test for selective allele frequency shifts.
Research sample	The lake and stream populations of Gasterosteus aculeatus chosen for the experiment occur in close neighborhood in the Lake Constance basin. They have been shown in previous research to be under divergent selection (Roesti et al. 2015, Nat. Commun. 6: 8767; Moser et al. 2016, J. Evol. Biol. 29: 711-719) for pelagic versus benthic life styles, and are therefore well suited for an experimental investigation of directional selection on natural genetic variation in action.
Sampling strategy	According to the poolSeq literature (Ferretti et al. 2013, Mol. Ecol. 22: 5561-5576; Gautier et al. 2013, Mol. Ecol. 22: 3766-3779), allele frequencies are estimated with adequate precision when using 30-50 individuals. Given we use > 200 individuals for the natural populations (and a read depth of around 210x), our initial genome scan must be considered of outstanding quality. Even for the survivor panel (N = 37), allele frequencies are still expected to be reasonably accurate, although this sample size was not under our control (we used all survivors available).
Data collection	Field sampling was conducted by DM, MR, DB TGL and MA with minnow traps. Sequence data were generated using Illumina sequencing of DNA pools to high read coverage.
Timing and spatial scale	Sampling of the natural populations occurred at our classical field sites (ROM lake site and NID stream site; Berner et al. 2010, Mol. Ecol. 19: 4963-4978; Moser et al. 2012, PLoS One 7: e50620) during the standard (spring) catch period - the precise dates are 13 April, 20 May and 14 June 2016 (Berner et al. 2017, J. Evol. Biol. 30: 401-411), thus yielding a well-balanced sample of individuals from across the entire breeding season. The experimental F2 hybrid fish were released as juveniles in late summer (Sep. 16, 2015), and re-captured approximately one year later (Aug. 30-31, 2016). This exposure to natural conditions allowed for selection to influence the survival of experimental fish.
Data exclusions	No data were excluded.
Reproducibility	This experiment cannot be replicated in a strict sense outside the focal stream, as ecological conditions are likely slightly different in different streams. Given the effort involved in this experiment (2.5 years of fish husbandry in the laboratory to obtain F2 hybrids), replication is not realistic. However, owing to a specific a priori prediction for the experimental outcome (evolution of candidate sites under selection, relative to genome-wide control sites), this study is conclusive as a stand-alone experiment. Data analysis is fully reproducible and all raw genomic data, code and input files necessary are provided as supplementary material.
Randomization	Sampling of the natural individuals occurred haphazardly from a larger collection of individuals taken for a previous experiment. Sampling of the reference individuals was random in a strict sense. Sampling of the survivors was exhaustive.
Blinding	Our experiment is genomic, hence based on allele frequencies. Allele frequencies cannot be guessed without sequencing. Blinding was thus neither possible nor necessary or feasible.

Did the study involve field work? ☒ Yes ☐ No

Field work, collection and transport

Field conditions	Sampling of the natural populations was performed during normal weather and temperatures (standard sampling conditions); these conditions have no relevance to the design of the experiment. The same applies to the release of the experimental F2 hybrid population, and the re-capture.
Location	Sampling of the natural populations occurred at standard sampling locations and micro-habitat, like in several earlier studies in this system. The locations are published, and the corresponding reference cited in the manuscript. For completeness, here the coordinates: Rom (lake), 47°33'22.5'' N, 9°22'48.25'' E. NID (stream): 47°33'29.25'' N, 9°16'42.38'' E.<br>The experimental field release site is a new location. The coordinates are 47°32'44.156" N, 9°13'23.631" E.
Access and import/export	All field work was performed with permission by the Fisheries Authorities of the Canton of Thurgau (permit issued in April 2015).
Disturbance	The field work for this experiment caused minimal disturbance to the habitats and to the surrounding environment. During the re-capture, non-target fish species were released immediately.

Reporting for specific materials, systems and methods

We require information from authors about some types of materials, experimental systems and methods used in many studies. Here, indicate whether each material, system or method listed is relevant to your study. If you are not sure if a list item applies to your research, read the appropriate section before selecting a response.

Materials & experimental systems

n/a	Involved in the study	
☒	☐	Antibodies
☒	☐	Eukaryotic cell lines
☒	☐	Palaeontology
☐	☒	Animals and other organisms
☒	☐	Human research participants
☒	☐	Clinical data

Methods

n/a	Involved in the study	
☒	☐	ChIP-seq
☒	☐	Flow cytometry
☒	☐	MRI-based neuroimaging

Animals and other organisms

Policy information about studies involving animals; ARRIVE guidelines recommended for reporting animal research

Laboratory animals	No laboratory animals were used
Wild animals	Wild threespined stickleback (Gasterosteus aculeatus; lake and stream ecotypes) were caught in the field using minnow traps, and transported to the fish facility of the Zoological Institute, University of Basel. There they were used for artificial crosses to establish pure-bred lines of stream and lake, then crossed into F1 hybrids, and then into F2 hybrids. For anesthesia we used Koi Med sleep, following guidelines from the Veterinary Office of the Canton of Basel City. F2 hybrids were then released into the wild at our experimental stream site. All fish transport ocured in large, oxygenated tanks, without recorded mortality.
Field-collected samples	Fish husbandry occurred using standard methods widely used for this species. Standard tank volume was 55 liters, although for the f2 hybrid population, larger tanks were used too. Water quality was checked every other day and feeding occurred twice every day, using standard food for the species (artemia and bloodworms).
Ethics oversight	All laboratory work was approved by the Veterinary Office of the Canton of Basel City, Switzerland.

Note that full information on the approval of the study protocol must also be provided in the manuscript.
