# Supplementary material for: Genomic release-recapture experiment in the wild reveals within-generation polygenic selection in stickleback fish
Source: Nat Commun. 2020 Apr 21;11:1928. doi: 10.1038/s41467-020-15657-3 (PMC7174299; doi:10.1038/s41467-020-15657-3)
Supplement: Supplementary file 4 — Description of Additional Supplementary Files [file 41467_2020_15657_MOESM4_ESM.pdf]

Description of Additional Supplementary Files

File Name: Supplementary Data 1
Description: Description of the raw Illumina sequence data files. The first column specifies the NCBI Sequence Read Archive (SRA) accession number for each sample.

File Name: Supplementary Data 2
Description: file containing all allele frequency data, for the Reference and Survivor pools, for the 0.1% SNPs of higher AFD between Lake and Stream (N=1023). Input for extracting the target SNPs (N=126; Supplementary Software, code section F)

File Name: Supplementary Data 3
Description: file containing all allele frequency data, for the Reference and Survivor pools, for the 126 target SNPs, filtered for minor allele frequency (0.25), read depth (70), representation (35 individuals) and physical distance (50 kb). Output from Supplementary Software, code section F.

File Name: Supplementary Data 4
Description: file containing data for the Reference and Survivors for the resampling approach based on neutral SNPs with AFD range of 0 to 0.1 between the natural populations of stream and lake. Input for Supplementary Software, code section G.

File Name: Supplementary Data 5
Description: file containing data for the Reference and Survivors for the resampling approach based on neutral SNPs with AFD deviating no more than +/- 25% of the genome-wide median (range 0.1 to 0.17). Input for Supplementary Software, code section G.

File Name: Supplementary Data 6
Description: file containing the initial frequencies of the favorable allele at the loci under selection, drawn from empirical observation, used for the simulated selection analysis. Input for Supplementary Software, code section H and I.

File Name: Supplementary Software 1
Description: Compilation of all main code used for data analysis. Input files required for each analytical step are specified in the preambles of the corresponding codes, and provided as Supplementary Data.
